# Evidence of a Natural Hybrid Oomycete Isolated from Ornamental Nursery Stock

**DOI:** 10.3390/jof9060627

**Published:** 2023-05-29

**Authors:** Clara Benavent-Celma, Debbie McLaggan, Pieter van West, Steve Woodward

**Affiliations:** 1 Department of Plant and Soil Science, School of Biological Sciences, University of Aberdeen, Aberdeen AB24 3UU, Scotland, UK; s.woodward@abdn.ac.uk; 2 International Centre for Aquaculture Research and Development (ICARD), Aberdeen Oomycete Laboratory, Institute of Medical Sciences, University of Aberdeen, Foresterhill, Aberdeen AB25 2ZD, Scotland, UK; d.mclaggan@abdn.ac.uk (D.M.); p.vanwest@abdn.ac.uk (P.v.W.); 3 Environmental and Biochemical Sciences Department, The James Hutton Institute, Craigiebuckler, Aberdeen AB15 8QH, Scotland, UK

**Keywords:** oomycetes, interspecific hybridization, international plant trade, pathogenicity

## Abstract

The oomycete genus *Phytophthora* includes many plant pathogens important in agricultural and environmental systems. Natural interspecific hybridization has been reported several times in *Phytophthora*, and although the fundamental processes of interspecific hybridization and the consequences of subsequent ecological distribution are poorly understood, reports suggest some hybrids can infect a broader host range and display enhanced virulence compared to the putative parental species. During a survey carried out at the University of Aberdeen in 2014–2015, of oomycetes present in ornamental plants purchased via the internet, a batch of oomycete isolates remained unidentified, showing, in some isolates, features generally related to hybridization. The aim of this study was to determine whether hybridization events had occurred between endemic and introduced oomycetes, probably/possibly facilitated through the international plant trade. The list of isolates examined included a putative hybrid closely related to *Phytophthora cryptogea*. The putative hybrid isolate was further characterized, and pathogenicity were tests carried out on *Eucalyptus globulus*, using an isolate of *P. cryptogea* as a positive control. Cloning of ITS, COXI and β-tubulin genes resulted in different sequence versions of the putative hybrid isolate; after mapping and a polymorphism position comparison, it was concluded that the studied isolate contained genetic information from *P. cryptogea*, *P. erythroseptica*, *P. kelmanii*, *P. sansomeana* and *Phytopythium chamaehyphon*. A PCR-RFLP assay, a NEBcutter analysis and flow cytometry analysis (genomes ranged between 0.168 to 0.269 pg/2C) added further evidence of the hybrid nature of this isolate. The putative hybrid presented complex growing patterns ranging from rosaceous to chrysanthemum-like and had an optimum growth temperature of 25 °C. Although the putative hybrid produced visible symptoms of disease on *E. globulus* seedlings, assessment of the relative susceptibility of *E. globulus* to *P. cryptogea* and the putative hybrid indicated that *P. cryptogea* was significantly more virulent than the putative hybrid, based on mortality, disease severity and foliar symptoms.

## 1. Introduction

The most important oomycete plant pathogen genera affecting agricultural and horticultural crops, ornamental plants and forest ecosystems are *Phytophthora* and *Pythium* [1,2,3]. The international trade in ornamental plants has been greatly transformed since the 1970s, as a result of: improved propagation methods; the adoption of new shipping methods [4]; increased consumer demand for exotic plants and instant landscapes leading to nurseries producing higher volumes of a wider range of ornamental plants [3,5]. The conditions associated with growing high numbers of plants in nurseries, such as high plant density, monocultures; a plentiful supply of water; and, sometimes, poor nursery management practices including inadequate drainage are extremely favorable for the development and reproduction of plant pathogens such as oomycetes [6]. The international plant trade has been identified as one of the most common pathways for the introduction of plant pests and pathogens to previously naïve environments, with potted ornamental nursery stock currently being considered the most important pathway based on the numbers of alien species, such as *Phytophthora,* being introduced [5,7,8]. In addition, potential interspecific hybridization poses an additional threat to biosecurity with the ability of *Phytophthora* species to hybridize being reported to contribute to the emergence of new plant pathogens [9,10]. Hybrids can occur when two distinct *Phytophthora* species interact in soil, host tissue, or water and form interspecific progeny. The resulting new combination of traits from each parental species has been shown to alter host specificities and the hybrids were able to infect plants outside the host range of the parental species [11], causing disease on previously unaffected host plants [10,12,13,14,15].

Although thought to be rare in nature, there is evidence that natural interspecific hybrids occur in oomycetes [10]. The potential for hybrid formation among *Phytophthora* species established in laboratory studies has been confirmed by the detection of hybrids in agro-ecosystems. First detected in the south east of the UK on dying alders in the 1990s, the pathogen, thought to have emerged from *Phytophthora cambivora* and a species related to *Phytophthora fragariae* [16], spread rapidly within a few years through waterways and via infected nursery stock [4,16]. It is believed that the hybridization occurred under nursery conditions, where the two putative parental species were brought together inadvertently by human activities [16,17,18]. Compared to its putative parental species, the descendants have a novel host specificity [12,19].

Hybridization events in *Phytophthora* Clade 8b were reported by [20] through sequencing and cloning two nuclear (ITS and Ypt1) and two mitochondrial (COX1 and NADH1) loci in combination with DNA content estimation by flow cytometry. Regarding *Phytophthora* Clade 8a, during a study on the phylogenetic relationships between species in the *Phytophthora cryptogea* complex and related species, *Phytophthora erythroseptica* and *Phytophthora sansomeana*, 19 hybrid isolates with multiple polymorphisms in the nuclear sequences were observed [21]. In 2013 [22], the hybridization hypothesis was tested on numerous *Phytophthora* Clade 6 species (associated with riparian ecosystems in South Africa and Australia) that were either highly polymorphic or were unsequenceable; four different hybrid types, that presumably emerged from sexual recombination, were identified in this study, with physiological traits tending to resemble those of the maternal parents. Natural hybridization also appeared to be common among five closely related indigenous Clade 6 *Phytophthora* species isolated from waterways and riparian ecosystems in Western Australia [23]. The nuclear genes were biparental in most cases, and in all cases mtDNA was uniparentally inherited, indicating the formation of hybrids through sexual crosses. Two natural interspecific allopolyploid *Phytophthora* hybrid species classified under ITS clade 7a were identified in 2017 [10] in Taiwan and each of the new species showed distinct differences in morphological characteristics, breeding systems, cardinal temperatures, and growth rates compared to related species. A recent study looking to unravel hybridization identified and characterized hybrids in the genus *Phytophthora* by developing a strategy which combined genome-wide genetic fingerprinting and genome size assessment [24]. The application of an innovative strategy resulted in 16 new hybrid species being identified, including the first interclade hybrid.

The increasing body of literature on hybridization in *Phytophthora* and *Pythium* species underlines the importance for their detection. As suggested above, hybridization events are preceded by contact between parental species under appropriate conditions which allow somatic or sexual hybridization; such contact may be facilitated by human activities such as in nurseries, the international plant trade and managed landscapes, where contact between species is enabled and favored. Following hybridization, hybrids proceed with new evolutionary traits and selection pressures may consequently result in rapid spread, exclusion of progenitors from the niche, successful competition for resources and/or changes in virulence enabling colonization of new hosts [12]. Overall, interspecific hybridization events have been described in at least six of the 12 phylogenetic clades of *Phytophthora*, including Clade 1, Clade 2, Clade 6, Clade 7, Clade 8 and Clade 9. Therefore, detecting, identifying and characterizing new putative hybrids during oomycete surveys is clearly an important step in the development of efficient control and management strategies, and in improving understanding of plant diseases caused by oomycetes.

Quantification of oomycete diversity present in the roots and compost of hardy ornamental plants bought from nurseries, retailers and internet sellers, was undertaken in recent work at the University of Aberdeen (Aberdeen, UK) using both isolation and molecular analyses [25]. In the survey, several oomycetes isolates from plant aerial tissues, roots and compost remained unidentified due to diverse identification difficulties, including isolates showing abnormal patterns of enzyme slippage during PCR, additivity, double chromatogram peaks and unreadable sequences. Given that these characteristic features are common in hybrids, we hypothesise that these isolates are potential hybrids, and our objective is to analyze the potential hybrid origin of these isolates.

## 2. Material and Methods

### 2.1. Isolate Revival, Maintenance and Single Hyphal Tip Culture

An oomycete survey of ornamental nursery stock was carried out at the University of Aberdeen in 2015, where woody ornamental plants (99) were analyzed destructively [25]. Isolate P3 (O-398) was obtained from the substrate of a *Pinus mugo* plant, with no foliar symptoms; *Pythium dissotocum* was identified from this substrate as well, and *Phytopythium littorale* and *Pythium sterilum* were found on the roots of the same plant. In 2020, isolate P3 was revived after 3 years storage on agar at 12 °C; pure cultures were maintained on potato dextrose agar (PDA, 39 g L^−1^, Oxoid, Cambridge, UK).To further reduce the chances of cross contamination with other oomycetes, bacteria, opportunistic fungi or a mixture of strains, a single hyphal tip culture method was used for subculturing [26]. A 6 mm diam. plug from a fresh culture was placed in the center of 2% water agar (WA; 20 g /1 L, Oxoid, Cambridge, UK). The isolate was subcultured six times and incubated at 25 °C in the dark until cultures had approximately 3 cm diam. growth. Cultures and a dissecting microscope were placed in a sterile laminar flow cabinet; a few single hyphae at the edge of each culture were marked and a tip removed immediately before the last branching point using a sterile scalpel. Each single hyphal tip was then transferred to six-compartment Petri dishes, containing PDB (PDB, 24 g L^−1^, Oxoid, Cambridge, UK) and incubated in the dark at 25 °C for 7 days before proceeding with DNA extraction.

### 2.2. DNA Extraction and PCR

DNA was extracted from 50–100 mg washed and dried mycelium following the protocol of [27] and stored at −20 °C.

Five molecular markers were used with the primers shown in Table 1.

DNA amplification was carried out using Promega Go Taq G2 DNA Polymerase (Promega, Southampton, UK). The reaction mixture contained 2 ng DNA per reaction, 5 μL 5× buffer containing 1.5 mM MgCl_2_, 0.2 mM each dNTP, 0.5 μM final concentration of each primer and 1.25 U Taq polymerase. Final volume was made up to 25 μL with water. PCR reactions were performed using a Life Technologies Veriti Thermal Cycler (Thermo Fisher Scientific, Swindon, UK) with the following program: initial denaturation step (95 °C, 3 min); 35 cycles (94 °C, 30 s); annealing for 30 s at (55 °C for ITS and COXI primers, 53 °C for COXII and 61 °C for β-tubulin primers); elongation step (72 °C, 1 min). After amplification, a final extension step (72 °C, 10 min) was included. Five reactions were performed for the potential hybrid isolate (P3) to reduce the potential for PCR bias. PCR products and amplicon sizes were checked and purified as standard. Sequences were analyzed and assembled using Geneious and the consensus sequences compared to sequences in GeneBank using the Basic Local Alignment Tool (BLAST).

### 2.3. Interspecific Hybridization Testing

#### 2.3.1. Cloning

Isolate P3 showed double chromatogram patterns in ITS sequences, a feature typical of previously reported hybrids; thus, a cloning assay was carried to further elucidate this hybrid hypothesis.

Purified amplification products of ITS, COX I and β-tubulin gene subsequences of putative hybrid isolate P3 were cloned using the Promega pGEM-T Easy Vector System Kit (Promega, UK); T cloning is a subcloning technique that avoids the use of restriction enzymes and where amplified inserts are cloned into linearized vectors with complementary 3′ thymine overhangs. Ligation and transformation were carried out according to manufacturer’s protocol. Single white colonies were picked from the transformation plates, inoculated individually into 5 mL LB with ampicillin and incubated at 37 °C with shaking at 150 rpm overnight. A DNA plasmid prep (GeneJET Plasmid Miniprep Kit (Thermo Fisher Scientific, Swindon, UK)) following the manufacturer’s protocol and plasmid DNA was sequenced by Eurofins Scientific (Ebersberg, Germany). All sequences were analyzed with Geneious, with the main objective of detecting the presence of dimorphic positions; for each cloned region, the different cloning types (e.g., when a polymorphism is repeated as a pattern several times within the list of cloning sequences) were detected and grouped before building a reference map.

#### 2.3.2. PCR-RFLP (Restriction Fragment Length Polymorphism)

A PCR-RFLP assay was established to reveal polymorphic sequence positions in the P3 hybrid isolate for ITS, COX I, COX II and β-tubulin genes. Amplification products were digested using Taq I and EcoRV Restriction Enzymes (Promega, UK) using the supplier’s protocol. At least three PCR products were digested for each ITS, COX I, COX II and β-tubulin, and each digestion was repeated at least three times to uncover possible changes in the banding patterns. *Phytophthora cryptogea* E2 (New Machar, Scotland, University of Aberdeen Culture Collection, October 2013, accession number in progress, temporary: OQ430842) was used as comparison (*P. cryptogea* could be a parental species of P3). The experiment was assisted using the NEB Cutter online tool (http://nc2.neb.com/NEBcutter2/index.php, accessed on 27 April 2023), which provided visualization of expected banding patterns following NEB enzyme digestion of DNA sequence.

#### 2.3.3. Flow Cytometry (FC)

Estimation of DNA content in the putative hybrid P3 isolate was carried out. During cloning, it was determined that *P. cryptogea* could be one of the parental species of P3; thus, *P. cryptogea* E2 (University of Aberdeen culture collection isolate) was also used in this assay for comparison. The genome size of *Phytophthora cryptogea* had been recently analyzed using FC [24].

P3 and *P. cryptogea* E2 were subcultured in 90 mm diam. Petri dishes containing 5 mL pea broth for 6–10 days. The assay was carried out using four internal DNA reference standards: *Arabidopsis thaliana*, *Raphanus sativus*, *Glycine max* and *Brassica oleracea*, with genome sizes (pg) of 2C = 0.32, 2C = 1.02, 2C = 2.34, 2C = 0.598, respectively [32] A trial with an isolate of *Phytophthora infestans* 88,069 (Dundee, Scotland, University of Aberdeen culture collection, March 2014), was also carried out; although this isolate had not been analyzed by flow cytometry previously, some publications on *P. infestans* provided consistent results: 2C = 0.4–0.6 (pg) depending on the strain [32], 2C = 0.1–0.8 (pg) [24]. The assay included four biological replicates and three technical replicates; on each day at least two standards were used with each sample. Extraction of nuclei was carried out using the Cystain PI Absolute P kit (Sysmex, Norderstedt, Germany). Approximately 0.5 cm^2^ of young leaf tissue for plant samples, and 100 mg of fresh mycelia were chopped with a sterile scalpel for 60 s. on a Petri dish containing 100 µL of Nuclei Extraction Buffer, the mixture left to stand for 30 s. and an additional 500 µL of Nuclei Extraction Buffer added. After standing for 60 s. the suspension was filtered through a 15 µm filter. For each sample and replicate, an unstained control (tube containing only the filtered suspension), and a stained tube to which 2 mL of propidium iodide staining solution was added after the filtering step, were prepared. Samples were covered with aluminum foil and left for 2 h prior to use. Measurements were made using a BD Fortessa (Ian Fraser Cytometry Centre, University of Aberdeen, Aberdeen, UK) equipped with 7 lasers, emitting at a fixed wavelength of 488 nm. Fluorescence was recorded through 695/40 and 670/14 blue filters with constant amplification settings throughout the experiment. Data were analyzed using FlowJo v10.71 software. The following parameters were recorded for each sample: forward light scatter (FS), to estimate relative size of particles, side light scatter (SS), to estimate relative optical complexity of particles, relative fluorescence intensity of PI-stained nuclei (FL) and half peak coefficient of variation (CV) of the G0/G1 peak to estimate nucleus integrity and variation in DNA staining [33]. DNA content was calculated using the ratio of peak position of objective sample and standard reference applying the formula:


$$\begin{array}{l} Nuclear\;DNA\;content\left(\frac{pg}{2C}\right)\\ =\frac{G1\;nuclear\;Peak\;Objective\;Sample}{G1\;nuclear\;Peak\;Standard\;Sample}\times 2C\;DNA\;amount\;of\;Standard \end{array}$$

Genome sizes were recorded as pg DNA/2C in which 2C corresponds to the complete DNA content of the nucleus [34] and also in Mbp: the conversion from pg to Mbp was made by multiplying the DNA content in pg by 978 [24].

### 2.4. Characterization of Isolate P3

#### 2.4.1. Effect of Temperature on Growth Rate

Colony growth rates and viability were analyzed using 3 different growth media: (1) 10% clarified V8A, (2) PDA, and (3) malt extract agar (MEA; 50 g/L, Oxoid, Cambridge, UK). Cultures were incubated at 5, 10, 15, 20, 25, 30 and 40 °C. P3 agar plugs (six mm diam) from 5–7 d-old cultures grown on PDA were placed at the center of Petri dishes containing the different media and incubated at 20 °C (12 h, in the dark). The margin of initial colony growth was marked before transferring cultures to different temperatures. The diameter of each colony was measured daily for 10 days. The experiment was carried out in triplicate. After 10 days, cultures incubated at the highest and lowest temperatures were placed at 20 °C (24 h) to establish if growth could occur thus to check for viability [35,36]. Results were then compared with the published growth rates and optimum temperature of *P. cryptogea* [37].

#### 2.4.2. Hymexazol Sensitivity

Hymexazol, is often added to the PARP culture medium for the specific isolation of *Phytophthora*, and certain *Phytopythium* species, as it inhibits the growth of *Pythium* species; however, there are certain *Phytophthora* species which are sensitive to [1,38]. In [39] the effect of hymexazol on the linear extension of hyphae of a range of Oomycetes was examined and modelled, revealing close relationships between sensitivity to the fungicide and the current classification of the Oomycetes. Thus, the putative hybrid isolate P3, was tested for hymexazol sensitivity; as sensitivity of *P. cryptogea* to hymexazol has not been reported, *P. cryptogea* E2 was also tested. Following the protocol of [39], 6 mm diam. plugs from 5–7 day-old cultures of P3 and *P. cryptogea* E2 were subcultured to PDA, with and without hymexazol (50 µg mL^−1^.) and incubated at 20 °C. Six replicates were prepared, diameter growth measured, and mycelial characteristics observed over 10 days.

#### 2.4.3. Pathogenicity Tests Using Eucalyptus Globulus

*Eucalyptus globulus* plants were germinated using double autoclaved substrate (All Purpose Growing Medium, Sinclair, Ellesmere Port, UK) and incubated (18 °C to 20 °C: 12 h photoperiod (80.72 µmol m^−2^ s^−1^); 60% humidity.) in a phytotron and sprayed with water every two days for 4–6 weeks until young seedlings emerged.

Inocula of *P. cryptogea* E2 and isolate P3 were prepared following the protocols given in [40,41]. Isolates were subcultured to fresh PDA and incubated at 25 °C in the dark for 7 days. Six 5 mm^2^ plugs of each isolate were transferred to 150 mL glass jars containing 50 g millet grain (previously autoclaved for 60 min ×2, 24 h interval) and 35 mL 20% V8 broth (200 mL V8 juice, 800 mL of distilled water and 3 gCaCO3). Controls (20) were mock-inoculated with 5 mm^2^ sterile PDA plugs. Inoculated millet jars were shaken by hand every two days to ensure homogeneous distribution of inoculum growth and left in the dark at 25 °C for 15 days prior to plant-soil inoculation [42].

Plastic pots (1.5 L) were filled with All Purpose Growing Medium (Sinclair, UK), 20 g of inoculated millet grain with *P. cryptogea* E2, P3 or control inoculum added and gently mixed. Young healthy *Eucalyptus globulus* seedlings were transplanted to each pot and watered every two days until harvesting. Greenhouse conditions were 20–25 °C day–15–20 °C night, 12 h photoperiod (light/dark; 185 µmol m^−2^ s^−1^). There were 20 replicates per treatment.

##### Damping-Off Test Analysis

Three weeks after inoculation, plants were harvested, and number of dead/alive plants recorded giving ‘mortality rate’. Foliar symptoms and root rot were rated visually using a modified 0–4 disease severity scale [43,44]:

Foliar symptoms: 0 = foliage without visible infection symptoms; 1 = foliage yellowing; 2 = foliage yellowing and tip wilting; 3 = yellowing, tip and total foliage wilting; 4 = plant death.

Root rot disease severity: 0 = roots without visible infection or discoloration; 1 = roots with light discoloration and light root and stem rot; 2 = short roots with discoloration; 3 = short roots with severe discoloration and root rot; 4 = plant death.

After evaluation for visible infections and symptoms, roots were washed in tap water and root length, fresh and dry weight determined. Root length measurements were carried out with electronic digital callipers. Plants were weighed (wet weight and dry weight) on an analytical balance; plants were dried to constant weight in a Gallenkamp drying oven at 60 °C, generally for 24–48 h.

##### Data Analysis

Data: mortality (percentage dead plants); Root Disease Severity (RootDS); Foliar Symptoms; plant length (height, mm); fresh weight (g); dry weight (g) were analyzed using R (v 3.3.1; R Foundation for Statistical Computing, Vienna) with an Open-Source License.

Analysis 1 compared inoculated plants with control plants. Plant length (height), fresh weight and dry weight were considered continuous variables. Normality was examined using the Shapiro-Wilk Normality Test and adjusted by the Holm method, whereas homogeneity of variances was tested using the Levene Test with the mean as centre. When all groups followed a normal distribution and variances were equal, differences were analyzed using the Two Sample T-Test (student-*t* test) indicating “var. equal= TRUE” in the R command; when all groups followed a normal distribution, but not variance homogeneity, the differences were tested using the Welch Two Sample T-Test indicating “var. equal = FALSE” in the R command; when normality could not be assumed, the non-parametric Wilcoxon rank sum Test was used. The variable Mortality was binary, thus a “2-sample test for equality of proportions without continuity correction” (Pearson’s Chi-Squared test) was used. RootDS and Foliar Symptoms (0–4) variables were qualitative ordinal variables that could not be properly analyzed as continuous variables. Thus, data were analyzed using the Wilcoxon test to illustrate differences between control and inoculated plants.

Analysis 2 compared pathogenicity of *P. cryptogea* E2 and isolate P3. For the variables, plant height, fresh weight and dry weight, Normality and Homogeneity of Variances were tested as detailed above. When these hypotheses could not be assumed, the Kruskall–Wallis Pairwise comparison using the Wilcoxon rank sum test was used. When normality and homogeneity of variances were present, an ANOVA model was used, and, if significant differences were observed, a multiple comparison using Tukey Contrasts was applied. If data had a normal distribution but variances were not homogeneous, the Welch Test One-Way Analysis of means not assuming variances and Pairwise comparisons using *t*-tests with non-pooled SD were used. A 2-sample test for equality of proportions without continuity correction (Pearson’s Chi-squared test) was used to compare the effects of *P. cryptogea* E2 and isolate P3 on *E. globulus*, based on the variable ‘Mortality’. The other variables (Root DS and Foliar Symptoms) were analyzed using the Wilcoxon rank sum test.

In all cases a significance level of *p* < 0.05 was used.

## 3. Results

### 3.1. Cloning Analysis

BLAST searches of ITS sequences from proposed hybrid P3 suggested a close relationship with *P. cryptogea* as the nearest match, and *P. erythroseptica* as the second closest match, whereas BLAST against COXI indicated an even closer relationship with *P. cryptogea*.

Cloning was carried out using ITS 4_5 Alt, COXI_Levup_LevLo and β-tubulin 5_6 primers, in which the presence of dimorphic positions was found. For each cloned region, the different cloning types were detected and grouped before building a reference map. All cloned sequences, plus the original P3 isolate sequences, were then mapped with *P. cryptogea* MH136878.1 as reference, using the map as a reference tool in Geneious; additional closely related species were also added to the mapping reference comparison to analyze the similarities between the dimorphic positions and other potential parental species. The species chosen for the comparison list were those with high homology and percentage query cover when blasting P3 sequences in NCBI. Overall, 4 cloning types were identified and grouped for COXI and β-tubulin, whereas 8 cloning types were identified and grouped for the ITS region (Table S1).

COXI cloning analysis showed 15 variable polymorphic positions with the prevalence of Cloning Type 1 (50% of all clones), representing sequence information of *P. cryptogea* except in a few positions. Cloning Types 2 (10%), 3 (5%) and 4 (35%) were very similar to Type 1 and thus to *P. cryptogea*, with a few more polymorphism positions that did not correspond to *P. cryptogea*. It was noticeable that for all Cloning Types some of the dimorphic positions did not correspond to any of the species added as reference comparisons (*P. cryptogea*, *P. erythroseptica*, *P. persiana*, *P. kelmanii*).

Tubulin 5_6 cloning analysis resulted in the grouping of 4 different cloning types with the prevalence of Cloning Type 1 (57% of all clones). Tubulin 5_6 was the region showing the most polymorphic positions with exact position patterns with one of the compared species used in the map to reference *P. cryptogea*, *P. erythroseptica* and *Phy. chamaehyphon*. Cloning Types 1, 2 (14.29%) and 3 (14.29%) showed similarities predominantly with *P. cryptogea* and, when the nucleotide differed from *P. cryptogea,* it corresponded with the base composition of *P. erythroseptica*. Thus, Cloning Types 1, 2 and 3 produced different combinations of *P. cryptogea* and *P. erythroseptica* sequences, respectively, with polymorphisms at variable positions. Cloning Type 4, also representing 14.29% of the total, showed identical sequence composition to *Phy. chamaehyphon* at all polymorphic positions found, providing strong evidence that *Phy. chamaehyphon* was involved in the hybridization.

ITS cloning analysis resulted in 8 different cloning types with the prevalence of Cloning Type 3 (30.09% of all clones) which was identical to *P. cryptogea*. Cloning Types 1 (4.55%), 2 (13.64%), 4 (6.82%) and 5 (4.55%) were mainly identical to *P. cryptogea,* with the exception of isolated variable positions that could correspond to *P. erythroseptica, P. sansomeana* or *P. kelmanii*. In contrast, Cloning Types 6 (13.64%), 7 (9.09%) and 8 (13.64%) showed different combinations of polymorphic positions that corresponded mainly to *P. erythroseptica, P. sansomeana* and *P. kelmanii*, with lower similarities to *P. cryptogea*. Analyses of the results are shown and summarized in Table S1. PCR-Restriction Fragment Length Polymorphism was carried out using Taq I and EcoRV and interpreted using the NEB Cutter online tool. Different banding patterns between P3 and *P. cryptogea* E2 sequences were observed and also among P3 cloning sequences. Polymorphisms detected in P3 (*P. x cryptogea* related Hybrid) during cloning assays with COXI, TUBULIN 5_6 AND ITS are shown in detail in the supplementary material (Table S1).

### 3.2. PCR-RFLP (Restriction Fragment Length Polymorphism)

The presence of differential base pairs in the studied regions fragmenting at particular positions was also examined in a PCR-RFLP assay to: (a) Compare the banding patterns between P3 and *P. cryptogea* E2 in different regions. A different banding pattern in some of the regions would increase evidence that P3 has close similarities to *P. cryptogea* but is actually a different species; and (b) Observe different fragmenting patterns in P3, which would indicate a hybridization process with each banding pattern corresponding to a different parental species.

**Figure 1:**
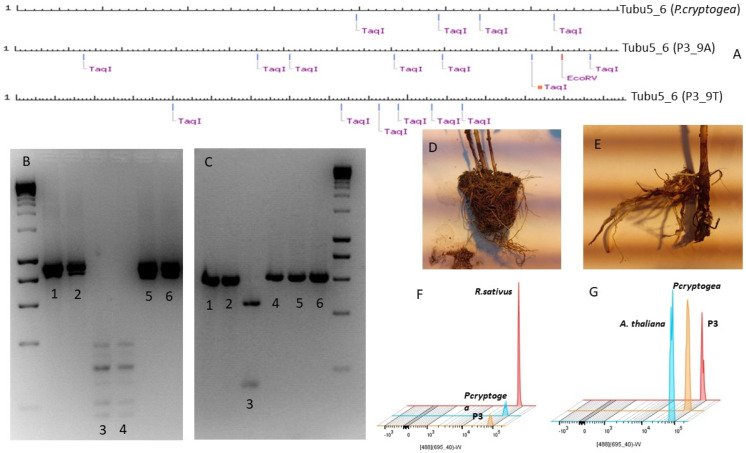
Visual comparisons between PCR-RFLP, Flow Cytometry and pathogenicity assays. (**A**) NEB cutter analysis. Differences in the banding pattern among *P. cryptogea*, and two different P3 cloning Tubulin PCR products (9A and 9T) when applying Taq I and EcoRV restriction enzymes. (**B**) PCR-RFLP assay ITS PCR products: (1) *P. cryptogea* PCR product, (2) P3 PCR product, (3) *P. cryptogea* PCR product with TaqI, (4) P3 PCR product with TaqI, (5) *P. cryptogea* PCR product with EcoRV, (6) P3 PCR product with EcoRV. (**C**) PCR-RFLP assay COXII PCR products: (1) *P. cryptogea* PCR product, (2) P3 PCR product, (3) *P. cryptogea* PCR product with TaqI, (4) P3 PCR product with TaqI, (5) *P. cryptogea* PCR product with EcoRV, (6) P3 PCR product with EcoRV. (**D**) Root system in a uninoculated control *E. globulus* plant. (**E**) Root system in inoculated plant with P3. (**F**) PI fluorescence histograms comparing the DNA peaks in radish, *P. cryptogea* and P3. (**G**) PI fluorescence histograms comparing the DNA peaks in *A. thaliana*, *P. cryptogea* and P3. In the histograms the x-axis represents the fluorescence intensity, and the y-axis represents the number of nuclei (counts), (488) (695/400-W: information representing the laser and filter used for the run.

The PCR-RFLP assay was carried out using Taq I and EcoRV as discriminatory enzymes cutting at 5′-TCGA-3′ and 5′-GATATC-3′, respectively. Interpretation was assisted using the NEB Cutter online tool to provide visualization of expected banding patterns after enzyme digestion of a DNA sequence. The results are shown in Figure 1A–C. Interestingly, NEBcutter showed different banding patterns (Figure 1A) not only between P3 and *P. cryptogea* E2 sequences (objective a), but also among P3 cloning sequences (objective b). Figure 1A–C shows visual results of both the PCR-RFLP and NEBcutter assay, and the number of cuts after NEB assay analysis are summarized in Table 2.

### 3.3. Flow Cytometry

DNA content was measured using FC for both P3 and *P. cryptogea* E2. Different plant and oomycete species (*P. infestans, B. oleracea, A. thaliana, R. sativus, G. max*) with published genome sizes available (either by FC or NCBI) were used as standards. The DNA content measurement by FC was estimated by the relative position of the peaks between the reference and the objective sample which is highly dependent on the selection of the standard, the extraction buffer, the chopping intensity and speed, and thus, the user extracting the nuclei. The results presented for this assay are considered an approximation of genome size and used for a relative comparison of P3 with *P. cryptogea*, to test the P3 x *P. cryptogea*-Hybrid hypothesis.

Genomes based on FC analyses (Table 3) ranged between 0.168 to 0.269 pg/2C, depending on the standard used, with two extreme and inconsistent results when using *R. sativus* and *G. max* (data not shown). Table 3 shows the results (±SD) obtained using each standard; *P. infestans* and *A. thaliana* appeared to be the most suitable standards with the most stable results. A histogram exhibiting the G_0_/G_1_ peaks produced for *A. thaliana* and *R. sativus* as standards is shown to illustrate the importance of a genome size similarity to achieve accurate results (Figure 1F,G). The most feasible and reproducible approximation was found with the two standards closer in genome size to the objective sample oomycetes, thus, *A. thaliana* and *P. infestans* (P3: 0.214–0.230 pg/2C, and *P. cryptogea* E2: 0.168–0.269 pg/2C).

### 3.4. Characterization of P3 Isolate, Recovered in the UK for the First Time

#### Colony Morphology, Growth Rate at Different Temperatures and Hymexazol Sensitivity

Colony growth rates and viability of P3 were analyzed to compare results with the published literature of growth rates and optimum temperature of *P. cryptogea* [37].

**Figure 2:**
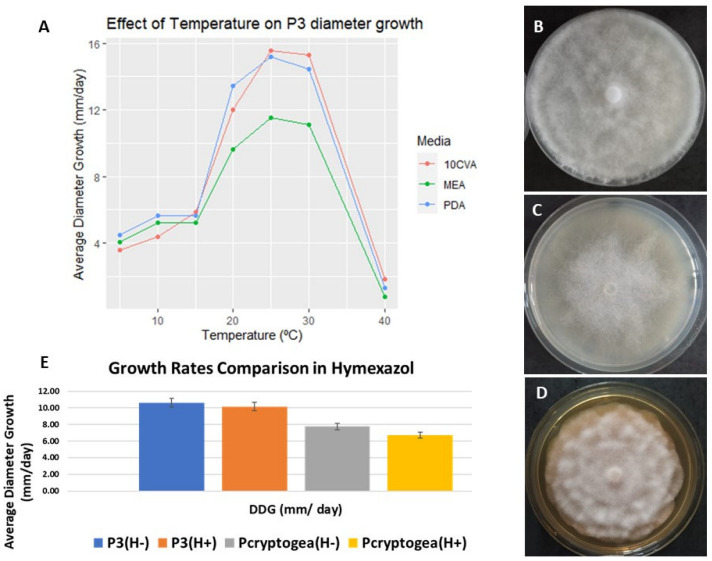
Growth characteristics of P3 (*P*. *x cryptogea*-related Hybrid). (**A**) P3 growth rates on 10%CV8, MEA and PDA at 5, 10, 15, 20, 25, 30 and 40 °C (average diameter calculated over 10 days). P3 growth after 7 days of incubation at 25 °C in the dark on PDA (**B**), C10%CV8 (**C**), MEA (**D**), (**E**) Growth rate comparison of P3 and *P. cryptogea* in PDA with and without hymexazol.

In 10% CV8, P3 (*P. x cryptogea*-related hybrid) produced clumped mycelia with submerged colony edges and a stellate pattern (1, B), a dispersed growth with a rosaceous pattern in PDA (1, C), and aerial, cottony growth showing a median pattern between rosaceous and petaloid in MEA (1, D). Isolate P3 had an optimum growth temperature range at approximately 25–30 °C in all media and remained viable after incubation at 5 or 40 °C. The fastest diameter growth rates measured were in 10% CV8 (15.55 mm/day) and PDA (15.17 mm/day), with a comparable slower growth rate in MEA (11.54 mm/day) (Figure 2A–D).

To determine hymexazol sensitivity, P3 and *P. cryptogea* E2 were grown in PDA with or without hymexazol: both isolates proved insensitive to this antibiotic (Figure 2E).

### 3.5. Pathogenicity Tests on Eucalyptus Globulus

An *in-planta* assay was conducted using *Eucalyptus globulus*, an easily obtained (seed) plant of known susceptibility to many *Phytophthora* species. Young seedlings of *E. globulus* were inoculated with isolate P3 and *P. cryptogea* E1 to evaluate the ability of P3 to infect woody plants. In this context, “pathogenicity” was defined as the ability of the pathogen to cause disease, and “virulence” as the extent of the pathology (disease) caused.

**Figure 3:**
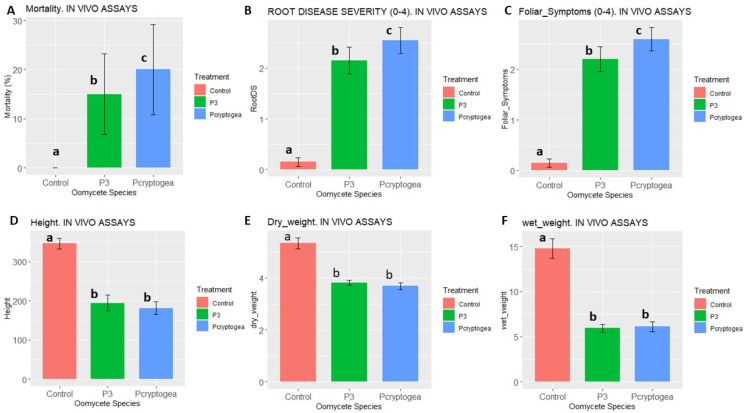
Pathogenicity indices of *Eucalyptus globulus* plants inoculated with P3 (*P*. *x cryptogea*-related Hybrid) and *P. cryptogea* E2 in comparison with controls. (**A**) Mortality (% of dead plants), (**B**) Root Disease Severity Index, and (**C**) Foliar Symptoms. (**D**) Plants Height (mm); (**E**) Plants Dry Weight (g); (**F**) Plants Wet Weight. Different letters above the bars indicate significant differences.

Both isolates P3 and *P. cryptogea* E2 showed pathogenicity to *E. globulus* plants (*p* < 0.05). In comparison to the control plants where no mortality was observed, inoculation with *P. cryptogea* E2 and P3 led to the death of 20% plants, (*p* = 0.03501) and 15% plants (*p* = 0.07172), respectively (Figure 3A). Similarly, in comparison to the controls, significant differences were observed in foliar symptoms for both *P. cryptogea* E2 (*p* = 1.991 × 10^−8^; and P3 (*p* = 3.776 × 10^−8^) (Figure 2C). Plant height (Figure 3D), fresh weight (Figure 3E) and dry weight (Figure 3E) were also significantly lower in both P3 and *P. cryptogea* E2 inoculated plants with compared to controls. Damping-off symptoms included root rot, poor vigour, wilted foliage, brownish lesions and rotting roots present in weak root systems with significant differences observed between inoculated plants and controls, as summarized in Table 4.

## 4. Discussion

Unidentified oomycetes isolated from ornamental plants during previous work at the University of Aberdeen [25] were subject to recovery protocols after long-term storage and to identification analysis, during which one of these isolates, isolate P3, showed characteristic features of a hybridization origin.

Advances in molecular techniques have enabled more in-depth examinations of phylogenetic relationships within various groups of microorganisms including oomycetes [45,46]. Among the available genetic markers used for oomycete phylogeny, the rapidly evolving, non-coding internal transcribed spacer region (ITS 1 and ITS 2) of ribosomal DNA has been one of the most common choices for high sequence variability and availability of primers [47,48]. ITS appears between two coding regions, 18S and 28S genes; another coding region, the 5.8S gene, occurs between ITS 1 and ITS 2. Sequence analysis of these non-coding regions has been employed to study intrageneric relationships within *Pythium* [48] and *Phytophthora* [10,49,50,51,52] and also the intergeneric relationships among these oomycetes [51]. Furthermore, advances on online resources as “IDphy” have recently been published. This online tool was developed to facilitate the correct identification of species of *Phytophthora,* allowing the user to work with molecular and morphological data, and emphasizing species of high economic impact and regulatory concern for the United States; that resource has also proven valuable for international researchers working with the genus *Phytophthora* [53].

The ITS region is sensitive to concerted evolution because it is located in rRNA genes that are repeated in tandem arrays of several hundred copies in each genome [20], however, the evolution of one gene does not represent the evolution of the entire genome [54]. Thus, it is necessary to include other independent genes in these analyses. Genes such as cytochrome oxidase I (COX I) and β-tubulin, which code for metabolic and structural proteins, respectively, are conserved and the alignment of these sequences is less ambiguous than the ITS region. Cytochrome oxidase (COX I mtDNA) sequence data was reported for utility in accurate species delimitation for oomycetes and suggested, together with the internal transcribed spacer (ITS), as a standard DNA barcode marker for oomycetes [28].

In this current work, following an in-depth analysis of ITS and COXI sequences and NCBI databases, sequencing of β-tubulin was carried out. Dimorphic double peaks in sequence chromatograms for ITS, COXI and β-tubulin, typical of hybrids, were observed. The sequence data indicated that *P. cryptogea* was most likely involved in the parentage of the proposed hybrid. Cloning of ITS, COXI and β-tubulin genes resulted in different sequence versions of P3 that were grouped into Cloning Types; overall, 4 cloning types were identified and grouped for COXI and β-tubulin, whereas 8 cloning types were identified and grouped for the ITS region.

After mapping and a polymorphism position comparison, it was concluded that P3 sequences contained genetic information from *P. cryptogea*, *P. erythroseptica*, *P. kelmanii*, *P. sansomeana* and *Phy. chamaehyphon*. It was noticeable that for all Cloning Types some of the dimorphic positions did not correspond to any of the species added as reference comparisons (*P. cryptogea*, *P. erythroseptica*, *P. persiana*, *P. kelmanii*). COXI cloning sequences would be expected to contain sequences of one of the putative parent species via maternal inheritance [55]. For COX1, although Cloning Type 1 represented predominantly information from *P. cryptogea*, some of the dimorphic positions of all Cloning Types (P55, P66, P725) did not correspond to either *P. cryptogea* or any of the species added to the reference list, suggesting somatic hybridization; that is, when different *Phytophthora* species are present in a common habitat, the zoospores can interact. Two interacting zoospores may fuse so that both nuclei are enveloped by one membrane. An example of this mechanism of hybridization was reported by [56] which described a new hybrid generated between *P. nicotianae* and *P. capsici.* Another, different mechanism of somatic hybridization can occur by heterokaryon formation through which hyphae of two species can fuse and a species hybrid is formed.

A similar occurrence was found in the ITS cloning sequence mapping, in which the presence of a polymorphism pattern failed to correspond to any reference species used in the map (Cloning Type 6, 7, and 8, P88). One possible scenario is that P3 may have resulted from a relatively ancient hybridization and the background of the events has been lost, thereby obscuring the whole hybridization background of the isolate [21,57]. The possibility that *Phy. chamaehyphon* was involved in the hybridization might point to a potential intergeneric hybridization. It is essential to explore this suggestion further to determine the extent of plasticity of evolution and hybridization in oomycetes.

The PCR-RFLP assay and the NEBcutter analysis added further evidence of the hybrid nature of isolate P3; the results of both assays showed that the banding patterns differed between *P. cryptogea* E2 and P3 sequences, and also among P3 cloning sequences. Nevertheless, interesting results were found from cloning ITS, COXI and β-tubulin, and given the results using COXII in the PCR- RFLP assay, it could have been interesting to carry out additional cloning with COXII to obtain more information regarding other possible parental species involved in the hybridization process. This work illustrated the difficulties and the importance of choosing and working with different DNA regions for species delimitation and examine potential hybridization processes; as shown in this study, species identification and hybrid detection can lead to uncertain or erratic results if only ITS and/or mitochondrial markers are used.

In plant pathology, detection, characterization and quantitation of pathogens are crucial for the development and application of control measures. A DNA content estimation of the incompletely characterized isolate P3 was carried out using FC with a range of reference standards. Similar results were obtained in replicate assays for P3 and *P. cryptogea* E2, except when using *G. max* or *R. sativus* as reference standards. Flow Cytometry analysis genomes ranged between 0.168 to 0.269 pg/2C, depending on the standard used. The results of DNA content estimation of isolate P3 were found to be reproducible when the standard used was close in genome size to the objective species (thus, *A. thaliana* and *P. infestans*) which we consider the most feasible approximation (P3: 0.214–0.230 pg/2C, and *P. cryptogea* E2: 0.168–0.269 pg/2C). These results were supported by the genome size measurements made using FC by [24], who estimated several *P. cryptogea* isolates that ranged from 0.221 to 0.224 pg/2C.

An unexpected result in this assay was the similarity in genome size between P3 and *P. cryptogea.* Based on other publications, a higher genome size of a hybrid (double or triple) would be expected, due to the hybridization background, which would have resulted in a larger number of loci compared to the number found in the progenitors. It remained clear that isolate P3 was not a new strain of *P. cryptogea*, as a rapid ITS sequencing and BLAST analysis might suggest. The similar genome size could be explained by similarities between some of the potential parental species (e.g., *P. cryptogea*, *P. erythroseptica*, *P. kelmanii*), however, with these findings, the finding of a *Pythium* species being involved in the hybridization process remains unclear; highly divergent progenitors share only a limited number of loci and may produce hybrids showing a substantial increase in numbers of loci [24]. This finding further emphasizes the need for stable and well-characterized oomycete internal standards for use in FC to achieve reliable and reproducible measurements.

Isolate P3 presented complex growing patterns, ranging from rosaceous to chrysanthemum-like, and had an optimum growth temperature of 25 °C. In terms of sensitivity to hymexazol, the results suggested a relative insensitivity of both P3 and *P. cryptogea* to this antibiotic/fungicide, with slower growth resulting but not complete inhibition. There are no specific reports of the effects of this fungicide on growth rates of the potential parental *Phytophthora* species listed above, although hymexazol has been reported to have a species/strain-specific effect [38].

Assessment of the relative susceptibility of *E. globulus* to *P. cryptogea* E2 and P3 and indicated that *P. cryptogea* E2 was significantly more virulent than P3, based on mortality, disease severity and foliar symptoms. In contrast, the variables height, or wet and dry weight of plants did not vary significantly between P3 and *P. cryptogea* E2, with such variables being significantly different for both isolates compared to controls. Although isolate P3 was less virulent than *P. cryptogea*, it was capable of producing disease symptoms in inoculated *E. globulus*. The reduced aggressiveness of *P. x cryptogea*-related hybrid P3 compared with *P. cryptogea* E2 towards *E. globulus* is consistent with the reported reduction in aggressiveness of some hybrids towards hosts of the parental species. However, it has been recognised that intra-specific hybridization between different oomycete species, divergent races of the same species, or populations from different ecological niches could generate pathogens with new host specificities and increased or decreased virulence [12,58].

In any *Eucalyptus* propagation programme, the nursery stage is a crucial point in terms of the risk of losing plants to pre-and post-emergence damping off, or root and collar rot disease [59]. *Phytophthora cryptogea* has been reported to cause damping off and root rot disease of *Eucalyptus* spp. in nurseries, among others *Phytophthora* and *Pythium* spp., such as *Phytophthora cinnamomi*, *P. citricola* and *Pythium anandrum* [60,61].

This work showing damping-off and root rot by *Phytophthora* species supports that of previous studies demonstrating that many *Pythium* and *Phytophthora* species cause damping-off and root rot in several ornamental and forest plants [62,63,64,65]. As a result of globalization and international plant trade and human movement, the chances of interspecific hybridization of oomycetes increases: this work presented evidence of the phytosanitary dangers posed by the international plant trade to the environment. Potted ornamental nursery stock is currently considered the most important pathway for the introduction of invasive species, evidenced by the number of reported *Phytophthora* and *Pythium* species outbreaks that have occurred in Europe due to the introduction of contaminated plants [3,7,8,66]. Therefore, the implications of the adaptative potential and pathogenicity of *Pythium* and *Phytophthora* hybrids deserve in depth examination.

Future research examining the functionality of polymorphisms found in the P3 isolate and other reported hybrids, i.e., whether these changes alter the function of a gene or set of genes, will contribute to the understanding of the mechanistic basis by which a particular polymorphism is associated with a specific change in aggressiveness, infection rate, or mode of action when attacking the host. It is clear from this work and previous publications, that hybridization in oomycetes poses a considerable threat in terms of the emergence of novel, potentially highly damaging taxa [10,11,12,13,16,23,24,34,56] and requires prompt further investigation. Further characterization of the isolate P3 would give valuable information on the morphology (sporangia, absence or presence of chlamydospores, and sexual phase) that would enable a comparison with *P. cryptogea* (characterized by the presence of nonpapillate sporangia, absence of chlamydospores, heterothallic oogonia and oospores production when paired with isolates of opposite mating types). In addition, inoculation of reported hybrid species on a wider range of ornamental plants (including *Pinus mugo,* the plant species from which P3 was originally isolated) under environmental conditions closer to those found in the field is required to better assess the threat hybrids pose to plant communities. It has been clear for some time that *Phytophthora* and *Pythium* pathogens are extremely adaptable [20], and mapping the relatively wide distribution of hybrids would be advantageous to further evaluate the environmental conditions required for these pathogens to spread, hybridize and infect novel hosts, thus enabling the development of improved control and management practices. Furthermore, this potential inter clade and inter genus finding is a possible first for *Phytophthora* hybrids. It is essential that we now look to establish this hypothesis beyond doubt.

## Figures and Tables

**Table 1:** Primers used in DNA amplification, cloning and PCR-RFLP assay.

Locus	Primer Name	Reference	Sequence 5′ to 3′
ITS ^a,b,c^	ITS 4 alt	[27]	TCCTCCGCTTATTGATATG
	ITS 5 alt		TGAAAAGTCGTAACAAGGTT
COX I ^a,b,c^	OomCoxI-Levup	[28]	TCAWCWMGATGGCTTTTTTCAAC
	Fm85mod		RRHWACKTGACTDATRATACCAAA
	OomCoxI-Levlo		CYTCHGGRTGWCCRAAAAACCAAA
COX II ^a,c^	COII-HF	[29]	GGCAAATGGGTTTTCAAGATCC
	COII-HR		CCATGATTAATACCACAAATTTCACTA
β-tubulin ^a,c^	TUBUF2	[30]	CGGTAACAACTGGGCCAAGG
	TUBUR1		CCTGGTACTGCTGGTACTCAG
β-tubulin ^a,b,c^	BT5	[31]	GTATCATGTGCACGTACTCGG
	BT6		CAAGAAAGCCTTACGACGGA

^a^ Primers used for amplification of DNA. ^b^ Primers used in the cloning assay. ^c^ Primers used in the PCR-RFLP assay.

**Table 2:** Resulting cuts when analyzing *P. cryptogea* and P3 sequences with the NEBcutter tool selecting Taq I and EcoRV as digestion enzymes.

Species	Primer (Enzyme)	
	ITS (Taq I)	ITS (EcoRV)
*P. cryptogea*	6 cuts	1 cut
P3	7 cuts	1 cut
	COX I (Taq I)	COX I (EcoRV)
*P. cryptogea*	2 cuts	Does not cut
P3	Varied: 1, 4, 5 (depending on cloning colony)	Does not cut
	TUBU5_6 (Taq I)	TUBU5_6 (EcoRV)
*P. cryptogea*	5 cuts	Does not cut
P3	Varied: 5, 6, 7, 11 cuts (depending on cloning colony)	Varied: 1 cut or doesn’t cut (depending on cloning colony)
	COX II (Taq I)	COX II (EcoRV)
*P. cryptogea*	1 cut	Does not cut
P3	Does not cut	Does not cut

**Table 3:** Genome size of isolates P3 (*P*. *x cryptogea* related hybrid) and *P. cryptogea* E2 estimated by flow cytometry. From the left column: the objectives sample analyzed; the standard used and its reference genome size in pg/2C; the genome size average expressed in pg/2C; and its standard deviation (SD); the genome size expressed in Mbp/2C; and its SD.

Species	Standard	Average (pg/2C)	Sd	Mbp/2C	Sd
P3	*Phytophthora infestans* (0.48 pg/2C)	0.230	0.0085	225.113	8.318
*P. cryptogea*		0.168	0.0082	164.593	8.026
P3	*Brassica oleracea* (0.598 pg/2C)	0.344	0.020	336.148	19.290
*P. cryptogea*		0.345	0.024	337.031	23.497
P3	*Arabidopsis thaliana* (0.32 pg/2C)	0.214	0.011	123.514	10.993
*P. cryptogea*		0.269	0.013	123.860	12.540

**Table 4:** In vivo tests comparing plant mortality, foliar symptoms, root disease severity, plant height and fresh and dry weights for *E. globulus* inoculated with *P. cryptogea*, P3 (*P*. *x cryptogea*-related Hybrid), (rows in bold) compared with controls. Data are means ± SD for each factor and the p-value (for Mortality, sample proportions are shown instead of the mean and SD). NS means *p* > 0.05, * means *p* ≤ 0.05, **** means *p* ≤ 0.0001. All statistical comparisons of control and inoculated plants carried out using Wilcoxon tests, except when stated otherwise.

	Mortality (%)	RootDS (0–4)	Foliar Symptoms (0–4)	Fresh Weight (g)	Dry Weight (g)	Height (mm)
*P. cryptogea* N = 20	0	0.15 ± 0.37	0.15± 0.37	14.80 ± 4.87	5.35 ± 0.95	343.09 ± 61.98
	**20**	**2.55 ± 1.15**	**2.60 ± 1.05**	**6.10 ± 2.54**	**3.69 ± 0.59**	**181.09 ± 74.21**
	*p* = 0.03501	*p* = 7.87 × 10^−8^	*p* = 1.991 × 10^−8^	*p* = 9.924 × 10^−9^	*p* = 1.374 × 10^−6^	*p* = 3.495 × 10^−9^
	(*)	(****)	(****)	(****)	(****)	(****)
P3 *P. x cryptogea-related hybrid* N = 20	0	0.15 ± 0.37	0.15± 0.37	14.80 ± 4.87	5.35 ± 0.95	343.09 ± 61.98
	**15**	**2.15 ± 1.18**	**2.20 ± 1.11**	**5.95 ± 1.93**	**3.82 ± 0.43**	**194.36 ± 92.16**
	*p* = 0.07172	*p* = 1.513 × 10^−7^	*p* = 3.776 × 10^−8^	*p* = 1.016 × 10^−10^	*p* = 6.008 × 10^−7^	*p* = 5.833 × 10^−5^
	(NS)	(****)	(****)	(****)	(****)	(****)

The relative susceptibility of *E. globulus* to the *P. cryptogea* E2 and P3 was also analyzed. A multiple comparison test between oomycete species used in inoculations was carried out: plants inoculated with *P. cryptogea* E2 had significantly greater root disease severity (*p* = 1.519× 10^−8^) and foliar symptoms (*p* = 1.453× 10^−9^) than plants inoculated with the newly identified hybrid P3. The variables wet weight, dry weight and height in plants inoculated with P3 or *P. cryptogea* were not significantly different.

## Data Availability

The authors confirm that the data supporting the findings of this study are available within the article (and/or) its supplementary materials.

## Supplementary Materials

The following supporting information can be downloaded at: https://www.mdpi.com/article/10.3390/jof9060627/s1, Table S1: Polymorphisms detected in P3 (*P. x cryptogea* related Hybrid) during cloning assays with COXI, TUBULIN 5_6 AND ITS.
