# Supplementary material for: Evidence of a Natural Hybrid Oomycete Isolated from Ornamental Nursery Stock
Source: J Fungi (Basel). 2023 May 29;9(6):627. doi: 10.3390/jof9060627 (PMC10302327; doi:10.3390/jof9060627)
Supplement: Supplementary file 1 [file jof-09-00627-s001.zip › jof-2398690-supplementary.pdf]

SUPPLEMENTARY MATERIALS

*Table S1: Polymorphisms detected in P3 (*P. x cryptogea *related Hybrid) during cloning assays with COXI, TUBULIN 5_6 AND ITS.*
A.1) COXI cloning types and percentage of each cloning type, comparison of variable nucleotide positions in the COXI cloning types using P.cryptogea MH136878.1 as a reference in the comparison map. B.1) β-tubulin cloning types and percentage of each cloning type (using BT 5 and 6 primers), comparison of variable nucleotide positions in the β-tubulin cloning types using P. cryptogea *MH760166.1as a reference in the comparison map. C.1) ITS cloning types and percentage of each cloning type, comparison of variable nucleotide positions in the ITS cloning types using* P. cryptogea *MH178331.1as a reference in the comparison map.*

**A.1) COX I MAP TO REFERENCE** *Phytophthora cryptogea*	P55	P498	P660	P689-P695	P705-P708	P725
P. cryptogea MH136878.1	G	A	T	TCGGTCA	TTAT	C
Type 1 (50%)	A	A	T	TCGGACA	ATCG	A
Type 2 (10%)	A	A	C	AATCACT	AGTG	T
Type 3 (5%)	A	A	T	CACTAGT	GCCG	A
Type 4 (35%)	A	A	C	TCGGTCA	ATCG/AGTT	A/T
Hybrid	A	M	T	----------	--------- -	
P. erythroseptica MH136882.1	G	T	T	TCGGTCA	TTAT	C
P. parsiana HM749282.1	G	T	T	TCGGTCA	TTAT	C
P. kelmanii KU220616.1	G	A	A	---------- ------		-

**B.1) TUBULIN 5_6 MAP TO REFERENCE** *Phytophthora cryptogea*	P26	P41	P59	P65	P71	P80	P92	P95	P107	P122	P125	P134	P155	P179	P185	P200
P. cryptogea MH760166.1	C	G	G	C	T	G	T	T	T	G	T	C	T	G	T	G
Type 1* (57%)	C	G	G	C	C	G	T	T	T	G	T	C	T	G	T	G
Type 2 (14.29%)	C	G	G	C	T	G	T	T	T	G	T	C	T	G	T	G
Type 3 (14.29%)	C	G	G	C	C	G	T	T	T	G	T	C	T	G	T	G
Type 4* (14.29%)	T	C	A	T	G	C	G	C	C	C	C	G	C	C	C	C
Hybrid (14.29%)	T	A	T	T	G	G	T	T	K	T	T	Y	T	G	Y	S
P.erythroseptica KX251896.1	C	G	G	C	C	G	T	T	T	G	T	C	T	G	T	G

Phy. chamaehyphon KJ595448.1	T	C	A	T	G	C	G	C	C	C	C	G	C	C	C	C
	P206	P209	P218	P227	P230	P239	P245	P254	P258	P269	P275	P278	P281	P290	P305	P320
P. cryptogea MH760166.1	T	C	C	C	G	T	G	G	T	T	G	G	G	C	T	T
Type 1	T	C	C	A	G	T	C	G	T	T	G	G	G	T	T	T
Type 2	T	C	C	C	G	T	G	G	T	T	G	G	G	C	T	T
Type 3	T	C	C	A	G	T	C	G	A	T	G	G	G	C	T	T
Type 4	C	G	G	G	C	C	G	C	T	C	C	C	C	C	C	C
Hybrid	T	Y	C	A	R	-	-	R	W	W	R	G	G	-	T	T
P.erythroseptica KX251896.1	T	C	C	A	G	T	C	G	T	T	G	G	G	T	T	T
Phy. chamaehyphon KJ595448.1	C	G	G	G	C	C	G	C	T	C	C	C	C	C	C	C
	P323	P329	P335	P338	P344	P366	P368	P374	P377	P407	P422	P431	P440	P446	P447	P449
P. cryptogea MH760166.1	T	C	G	C	T	T	G	G	C	G	C	T	T	T	T	A
Type 1	T	C	G	C	T	C	G	G	C	G	C	T	T	T	T	A
Type 2	T	G	G	C	T	T	G	G	C	G	C	T	T	T	T	A
Type 3	T	C	G	C	T	C	G	G	C	G	C	T	T	T	T	A
Type 4	C	G	C	G	C	C	C	C	G	A	T	G	C	C	C	G
Hybrid	Y	M	G	G	T	S	S	R	C	-	C	W	T	T	T	A
P.erythroseptica KX251896.1	T	C	G	C	T	C	G	G	C	G	T	G	C	C	T	A
Phy. chamaehyphon KJ595448.1	C	G	C	G	C	C	C	C	G	A	T	G	C	C	C	G
	P452	P458	P461	P464	P470	P485	P497	P515	P554	P590	P608					
P. cryptogea MH760166.1	T	G	G	G	C	G	T	G	T	C	C					
Type 1	T	G	G	G	C	G	C	G	T	T	T					
Type 2	T	G	G	G	T	G	T	G	T	C	T					
Type 3	T	G	G	G	C	G	C	G	T	T	T					

Type 4	G	A	C	C	T	C	C	C	-	-	-		
Hybrid	T	R	Y	G	C	A	T	G	T	Y	-		
P.erythroseptica KX251896.1	T	G	G	G	C	G	C	G	T	T	T		
Phy. chamaehyphon KJ595448.1	G	A	C	C	T	C	C	C	G	C	C		
**C.1) ITS MAP TO REFERENCE** *Phytophthora cryptogea*	P52	P78	P88	P104	P107	P212	P530	P632	P681	P695	P759	P764	778
P. cryptogea MH178331.1	C	T	T	G	T	A	A	G	T	A	T	T	C
Type 1 (4.55%)	C	T	T	G	T	G	A	A	T	A	T	T	C
Type 2 (13.64%)	C	T	T	G	T	A	A	A	T	A	T	T	G
Type 3 (34.09%)	C	T	T	G	T	A	A	G	T	A	T	T	C
Type 4 (6.82%)	C	T	T	A	T	A	G	G	T	A	T	T	C
Type 5 (4.55%)	T	T	T	G	T	A	A	G	T	A	T	T	G
Type 6 (13.64%)	C	A	C	G	T	A	A	A	T	A/G	C	C	C
Type 7 (9.09%)	C	A	C	G	T	A	A	G	T	A	T	T	C
Type 8 (13.64%)	C	A	C	G	C	A	A	A	T/C	A	C	C	C
Hybrid	-	-	-	-	-	A	A	R	T	A	Y	Y	C
P. erythroseptica KJ755119.1	C	A	T	G	T	A	A	G	T	A	C	T	C
P. sansomeana MF149917.1	C	A	T	G	T	A	A	G	T	A	T	C	C
P. kelmanii MN540003.1	C	A	T	G	C	A	A	A	T	A	C	C	C

* Type 4 (Tubulin 5_6), almost identical to Type 1 (Tubulin 5_6) but with polymorphism patterns shown to be closely related to *Phytopythium chamaehyphon*, the pattern is constant along the whole map to reference analysis
